# Utilization of infectious clones to visualize *Cassava brown streak virus* replication in planta and gain insights into symptom development

**DOI:** 10.1007/s11262-019-01697-5

**Published:** 2019-08-06

**Authors:** Katie R. Tomlinson, Susan E. Seal, Andy M. Bailey, Gary D. Foster

**Affiliations:** 1 School of Biological Sciences, University of Bristol, 24 Tyndall Ave, Bristol, BS8 1TQ UK; 2 Natural Resources Institute, University of Greenwich, Central Avenue, Chatham Maritime, UK

**Keywords:** *Cassava brown streak virus*, *Ugandan cassava brown streak virus*, Cassava, Food security, Infectious clones

## Abstract

Cassava brown streak disease (CBSD) is a leading cause of cassava yield losses across eastern and central Africa and is having a severe impact on food security across the region. Despite its importance, relatively little is known about the mechanisms behind CBSD viral infections. We have recently reported the construction of *Cassava brown streak virus* (CBSV) and *Ugandan cassava brown streak virus* (UCBSV) infectious clones (IC), which can be used to gain insights into the functions of viral proteins and sequences associated with symptom development. In this study, we perform the first reporter gene tagging of a CBSV IC, with the insertion of green fluorescent protein (GFP) sequence at two different genome positions. *Nicotiana benthamiana* infections with the CBSV_GFP ICs revealed active CBSV replication in inoculated leaves at 2–5 days post inoculation (dpi) and systemic leaves at 10–14 dpi. We also constructed the chimera CBSV_UCP IC, consisting of the CBSV genome with a UCBSV coat protein (CP) sequence replacement. *N. benthamiana* infections with CBSV_UCP revealed that the CBSV CP may be associated with high levels of viral accumulation and necrosis development during early infection. These initial manipulations pave the way for U/CBSV ICs to be used to understand U/CBSV biology that will inform vital CBSD control strategies.

**Electronic supplementary material:**

The online version of this article (10.1007/s11262-019-01697-5) contains supplementary material, which is available to authorized users.

## Introduction

Cassava brown streak disease (CBSD) is currently having a severe impact on cassava production in East and Central Africa [1]. Across Africa, cassava is the second most important crop in terms of *per*-*capita* calories consumed [2]. Cassava produces carbohydrate rich storage roots that are consumed by the grower or sold at markets to generate income [3]. Unlike other staple food crops, cassava can be harvested throughout the year, grows on marginal soil and tolerates unpredictable rainfall [3] and so is predicted to provide opportunities for climate change adaptation in Africa [4]. CBSD symptoms include storage root necrosis, radial root constrictions, foliar chlorosis, brown streaks on stems and stunting [5].

CBSD is caused by at least two related viral species: *Cassava brown streak virus* (CBSV) and *Ugandan cassava brown streak virus* (UCBSV) collectively termed U/CBSVs, which belong to the family *Potyviridae* of the *Ipomovirus* genus [6–8]. U/CBSVs are reported to be semi-persistently transmitted by *Bemisia tabaci* (whitefly) [9], however, relatively little is known about their interactions with insect vectors. U/CBSVs have single stranded, positive sense RNA genomes, which are translated as polyproteins and autocatalytically cleaved at specific cleavage sites by the viral proteinases: P1 and NIa-Pro into ten mature peptides, and an additional P3N-PIPO protein is produced by a +2 ribosomal frame-shift in the P3 sequence [10, 11].

CBSV and UCBSV produce distinct symptom types in different cassava cultivars and indicator hosts; CBSV tends to cause more severe necrosis and accumulates to higher titers, compared with UCBSV [6, 12–14]. In cassava, CBSV tends to cause feathery chlorosis along vein margins, which develops into chlorotic blotches, whereas UCBSV tends to cause circular chlorotic blotches between veins [6, 12–14]. The genome sequences responsible for symptom and titer differences between CBSV and UCBSV infections remain uncharacterized.

CBSV and UCBSV genomes typically share ≈ 70% nucleotide sequence similarity [6]. However, specific regions of CBSV and UCBSV genomes share lower homology. For instance, CBSV coat proteins (CP) are variable at their N’ ends and unlike UCBSV CPs, the N’ terminals of CBSV CPs typically contain a nine amino acid insertion and encode the highly conserved DAG motif [6, 15–17] which is involved with aphid transmission in a number of potyviruses. The N’ terminus of potyviral CPs tend to be exposed on the virion surface and are composed of disordered amino acid sequences to enable interactions with a wide range of viral and host proteins [18–20]. Therefore, sequence differences in U/CBSV CPs may be associated with differences in CBSV and UCBSV infections, which requires further investigation.

Despite the importance of U/CBSVs, relatively little is known about their fundamental molecular biology and sequences associated with symptom development. Progress has been hampered by the lack of U/CBSV infectious clones (IC) due to sequence instability during propagation in *Escherichia coli.* We have recently constructed three U/CBSV ICs, which can be manipulated to enable characterization of viral sequences involved with symptom development, pathogenicity, host-range, host interactions, movement and vector transmission [21]. In this study, we performed the following manipulations of the CBSV_Tanza IC: (1) insertion of the marker gene green florescent protein sequence and (2) construction of a chimera IC containing a UCBSV CP sequence replacement. This is the first report of a reporter tagged CBSV IC being used to characterize infections. Tagged U/CBSV ICs could be used to: (1) characterize viral sequences associated with viral replication and movement; (2) determine whether CBSV and UCBSV co-infect cells during mixed infections and (3) identify vector-binding sites. Meanwhile, chimeric U/CBSV ICs could be used to characterize genome sequences associated with differences in symptom development and viral accumulation during CBSV and UCBSV infections.

## Materials and methods

### Viral infections

Infected plant material and viral ICs were used under the DEFRA license No. 51045/197610/2 and handled according to Brewer et al. [22]. *Nicotiana benthamiana* plants were grown in growth cabinets 28 °C with a 16 h/8 h: light/dark cycle. Plants were agroinfiltrated with CBSV_Tanza IC plasmids according to the protocol described in Duff-Farrier [21] and mechanically inoculated with U/CBSV infected plant material (4 g) according to Ogwok et al. [23].

### Modification of the CBSV_Tanza IC

**Fig. 1:**
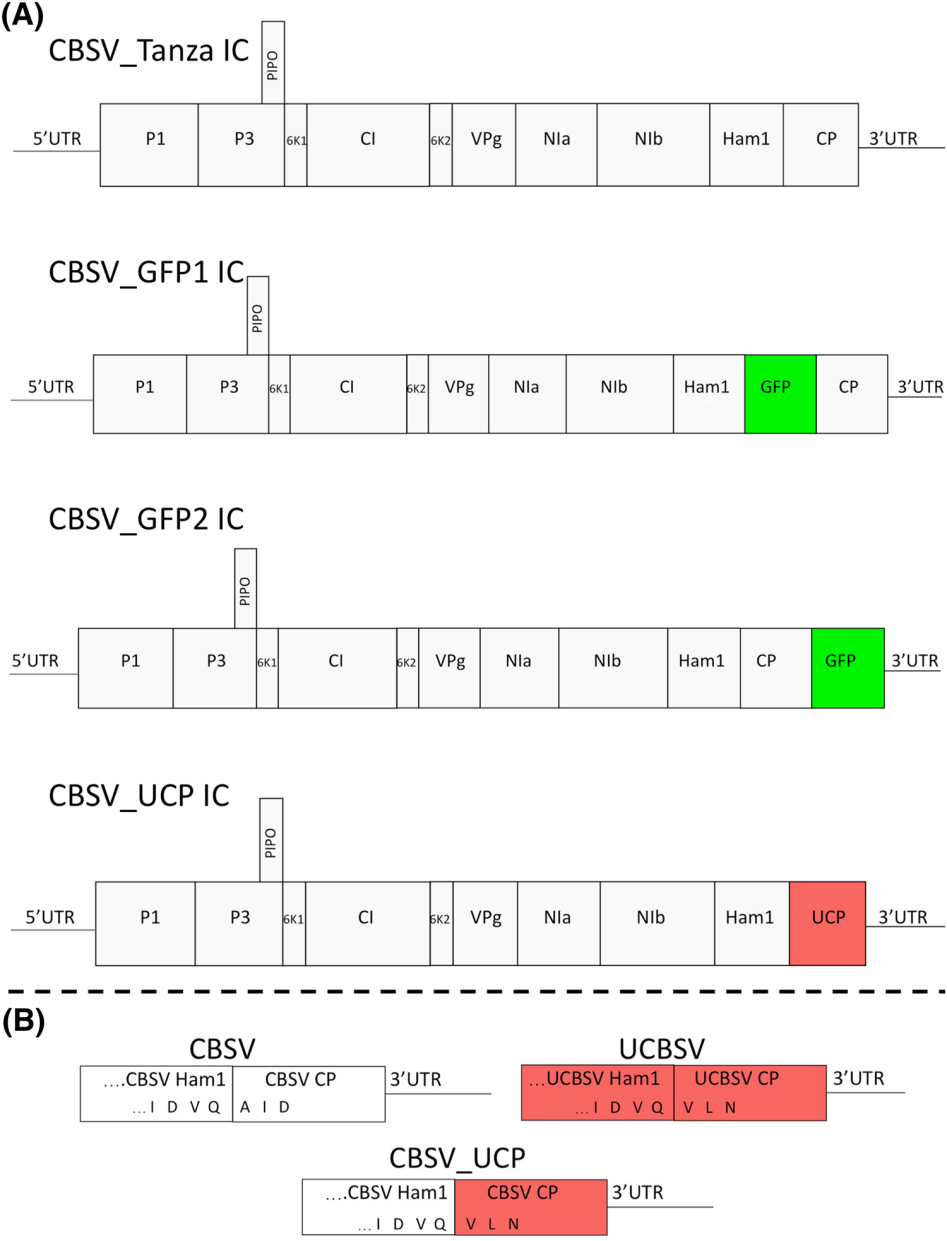
**a** Genome structures of the CBSV_Tanza infectious clone (IC) and modified ICs: CBSV_GFP1, CBSV_GFP2 and CBSV_UCP. The CBSV_Tanza genome consists of: 5′ and 3′ untranslated regions (UTR); proteinases (P1 and NIa); the third protein (P3); P3 N-PIPO produced through +2 frame-shift; 6-kDa proteins (6K1 and 6K2); cylindrical inclusion protein (CI); viral genome-linked protein (VPg); main viral proteinase (NIa); RNA-dependent RNA replicase (NIb); Ham1-like proteins and coat protein (CP). CBSV_GFP1 consists of the CBSV Tanza genome with GFP sequence inserted between the Ham1 and CP regions; CBSV_GFP2 contains GFP sequence between the CP and 3′UTR and CBSV_UCP consists of the CBSV_Tanza genome with a UCBSV Kikombe CP (red) replacement. **b** Schematics of the proteolytic cleavage sequences between Ham1 - CP peptides of the CBSV, UCBSV and CBSV_UCP polyproteins. In the CBSV Tanza polyprotein, Ham1- CP peptides are cleaved at the proteolytic cleavage site: I-D-V-Q-/-A, whereas in the UCBSV Kikombe genome they are cleaved at: I-D-V-Q-/-V. To enable release of the UCBSV CP from the CBSV polyprotein, the I-D-V-Q-/-V proteolytic cleavage sequence was included between the CBSV Ham1 and the UCBSV CP

Modifications of the CBSV_Tanza IC were performed using homologous yeast recombination according to Duff-Farrier [21]. Schematics for the construction of the modified CBSV_Tanza ICs are provided in Figs. S1, S2 and primers are provided in Table S1. Briefly, the CBSV_Tanza IC was digested with restriction enzymes that cut either side of the insertion site and digested CBSV_Tanza IC was then gel purified. Three overlapping PCR fragments were amplified using a high-fidelity polymerase and primers that add 30 base pairs of homologous sequence to the 5′ and 3′ ends. The digested CBSV_Tanza IC and PCR fragments were then transformed into yeast according to: Gietz [24]. Yeast plasmids were extracted and transformed into electrocompetent *E. coli* TOP10 cells. To confirm construction, the modified CBSV_Tanza ICs were analyzed by restriction digestion, PCR and Sanger sequencing. The genome structures for the modified CBSV_Tanza ICs are provided in Fig. 1 and sequences are available on the NCBI database under the accession numbers MK409379 (CBSV_GFP1), MK409380 (CBSV_GFP2) and MK409381 (CBSV_UCP).

For CBSV_GFP1/2 ICs proteolytic cleavage sites were included flanking GFP to enable cleavage of from the CBSV polyprotein. In CBSV_GFP1, the Ham1-CP cleavage site: I-D-V-Q-/A was added either side of GFP, whereas in CBSV_GFP2, the NIa–NIb cleavage site: I-S-V-Q-/A was added to the 5′ of the GFP sequence and no cleavage site was necessary at the 3′ end, as GFP is the last peptide in the polyprotein (Fig. S1). Proteolytic cleavage site sequences were designed so that third base of each codon was modified to a different nucleotide to encode the same amino acid but reduce spurious homology with the corresponding cleavage sequence elsewhere in the genome. This was an attempt to reduce homologous recombination between the two cleavage sequences, which could result in deletion of the GFP sequence. GFP sequence was amplified by reverse transcription PCR (RT-PCR) on the *N. benthamiana* 16c line (kindly donated by Professor Sir David Baulcombe), which constitutively expresses GFP [25].

For CBSV_UCP, the genome structure was designed to consist of the CBSV Tanza genome with a UCBSV Kikombe CP replacement (Fig. 1). In the CBSV Tanza genome, the Ham1 and CP peptides are separated by the proteolytic cleavage site: I-D-V-Q-/-A, whereas in the UCBSV Kikombe genome they are separated by the sequence: I-D-V-Q-/-V. Therefore to enable release of the UCBSV CP from the CBSV polyprotein, CBSV_UCP was designed to have the I-D-V-Q-/-V sequence between the CBSV Ham1 and the UCBSV CP (Fig. 1). As the CP is the last encoded peptide before the 3′UTR sequence, there was no need to clone a proteolytic cleavage sequence at the 3′ end of the UCBSV CP sequence. The UCBSV Kikombe CP sequence was amplified from the UCBSV_Kikombe IC [21] (NCBI: KX753357). Unfortunately, sequence instability issues during propagation in *E. coli*, prevented the use of the UCBSV_CCP IC, consisting of a UCBSV Kikombe genome with CBSV Tanza CP replacement.

### Detection and quantification of viral infections

To detect viral infections, RT-PCR was performed on 1 μg of plant RNA using a First Strand cDNA synthesis kit and an oligo d(T)18 primer. Viral specific primers were then used to amplify RT-PCR fragments from the cDNA, and RT-PCR fragments were then Sanger sequenced. Quantification of viral transcript abundance was performed using quantitative real-time PCR (qPCR) on diluted cDNA. To amplify CBSV transcripts, primers were used which target the CBSV CP and the F-Box gene was used as the endogenous reference gene, as it is reported to show relatively stable expression during viral infections of *N. benthamiana* [26]. All qPCR primer sequences are provided in Table S2. Relative CBSV CP transcript abundance was calibrated using three non-inoculated plants, according to the 2^−ΔΔCt^ method [27]. The abundance of U/CBSV CPs was measured using the U/CBSV-specific Triple Antibody Sandwich Enzyme Linked Immunosorbent Assay (TAS-ELISA) kit (DSMZ). U/CBSV CP titers in test plants were calculated relative to titers in the positive control sample provided with the kit (100%). Negative controls were included: where no sample was added to the extraction buffer and samples from non-inoculated plants. A cut-off threshold was calculated as: cut-off = (Mean OD 405 nm values for non-inoculated controls + 3 standard deviations). We deemed that viral titers in plant samples with OD 405 nm values below this threshold could not be accurately quantified.

### Quantitative symptom assessment

Plants were assessed for disease symptoms and rated according to the following scoring system, adapted from Ogwok et al. [23]: 1 = no symptoms; 2 = necrosis/chlorosis on agroinfiltrated leaf; 3 = mild systemic necrosis/chlorosis; 4 = severe systemic necrosis/chlorosis and 5 = plant death.

### Microscopy

Green fluorescence was visualized in whole leaves using a Leica CL5 Fluorescence microscope with the GFP2 filter (480/40 nm excitation and 510 nm barrier) and a confocal microscope (Leica TCS SP5) was used to visualize GFP in individual cells.

## Results

### Visualization of CBSV_GFP1/2 replication and movement

**Fig. 2:**
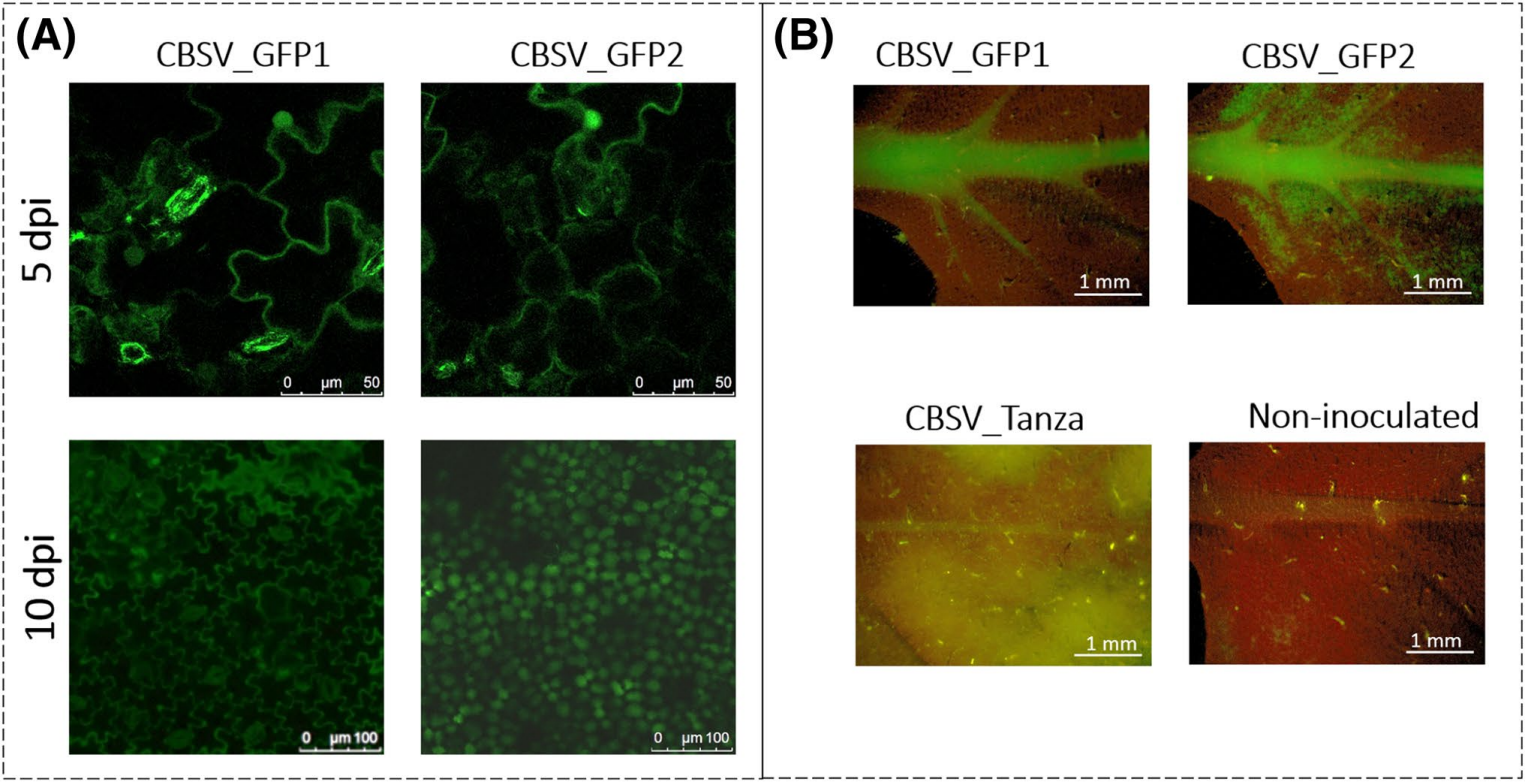
GFP expression during CBSV_GFP1/2 infections of *Nicotiana benthamiana*, indicating CBSV replication and systemic movement in planta. **a** Confocal microscopy images of green fluorescence (490–505 nm) in the epidermal cells of *N. benthamiana* leaves agroinfiltrated with CBSV_GFP1 and CBSV_2 at 5 dpi and in the epidermal and mesophyll cells of upper systemic leaves at 10 dpi in *N. benthamiana* plants infected with CBSV_GFP1 and CBSV_GFP2. **b** Wide-field microscope images of green fluorescence in the vascular system and leaf lamina of upper systemic *N. benthamiana* leaves infected with CBSV_GFP1 and CBSV_GFP2 at 14 dpi, compared with no green fluorescence in the upper systemic leaves of *N. benthamiana* plants agroinfiltrated with CBSV_Tanza IC and non-inoculated plants

Five-weeks-old *N. benthamiana* plants were agroinfiltrated with the unmodified CBSV_Tanza IC, the CBSV_GFP1 IC or the CBSV_GFP2 IC. Infections were performed in three repeat experiments, which produced the following consistent results. During both CBSV_GFP1 and CBSV_GFP2 infections, GFP was visible in epithelial and mesophyll cells of agroinfiltrated leaves at 2–5 days post inoculation (dpi), the vascular system of upper leaves at 7 dpi and epithelial and mesophyll cells in the lamina of upper systemic leaves at 10–14 dpi (Fig. 2). After 18–21 dpi, GFP was no longer visible. RT-PCR (Fig. S3) and amplicon sequencing (Figs. S4, S5) confirmed the presence of GFP sequence in the upper leaves of CBSV_GFP1/2 infected plants at 10 dpi. This demonstrates that marker gene tagged CBSV ICs can be used to visualize CBSV replication.

Compared with the highly necrotic symptoms that developed during infections with the unmodified CBSV_Tanza IC, CBSV_GFP1 and CBSV_GFP2 infections were asymptomatic (Fig. S6). QPCR analysis also demonstrated that viral transcripts were dramatically lower in CBSV_GFP1/2 infected plants, compared with unmodified CBSV_Tanza infections (Fig. S7). This suggests that the insertion of the GFP sequence has a large effect on CBSV symptom development and viral accumulation. Attempts to use CBSV_GFP1/2 infected leaf material to mechanically back-inoculate *N. benthamiana* and cassava plants were unsuccessful, which is likely to be due to low-viral titers. CBSV transcripts containing a complete deletion of GFP were detected in the upper systemic leaf material of 25% of CBSV_GFP1 and CBSV_GFP2 infected plants, at 15 dpi using RT-PCR (Fig. S3) and amplicon sequencing (Figs. S8, S9). This demonstrates that GFP can be precisely deleted from modified CBSV during *N. benthamiana* infections.

### Association of CBSV Tanza CP with symptom development and viral accumulation

To determine whether the CBSV Tanza and UCBSV Kikombe CP sequences are representative of their respective species, a phylogenetic tree was built with 19 CBSV and 23 UCBSV CP amino acid sequences. This revealed that that the CBSV Tanza and UCBSV Kikombe CP sequences cluster within their respective species clades and so should be relatively representative of their respective species (Fig. S10).

**Fig. 3:**
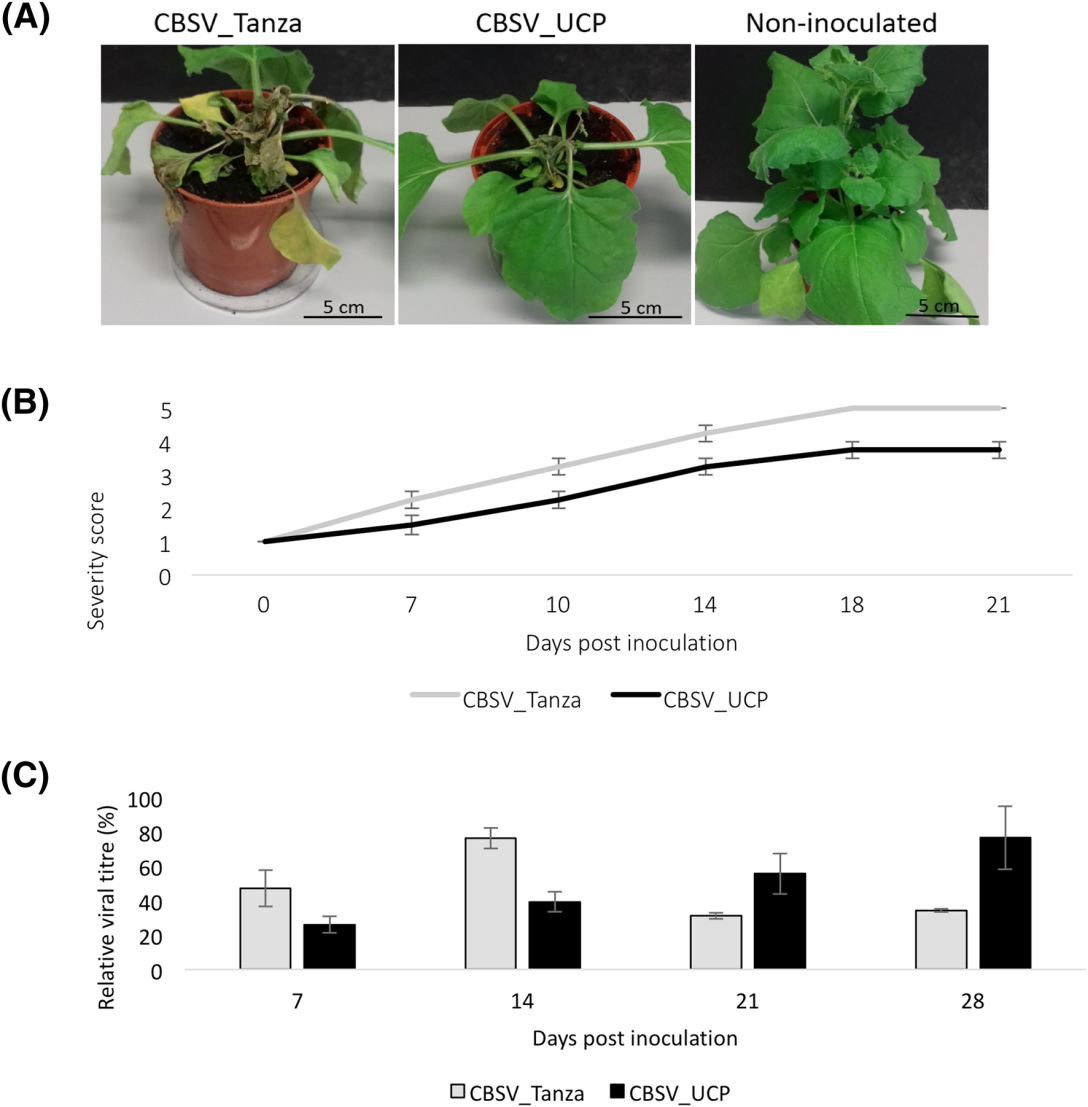
Characterization of differential symptom development and viral accumulation during *N. benthamiana* infections with CBSV_UCP, compared with unmodified CBSV_Tanza infections. **a***N. benthamiana* infections with CBSV_Tanza develop severe systemic chlorosis and necrosis, whereas infections with CBSV_UCP develop milder necrosis in upper systemic leaves, compared to asymptomatic non-inoculated plants. **b** Symptom severity scores throughout *N. benthamiana* infections with CBSV_Tanza and CBSV_UCP. Infections with CBSV_Tanza develop necrosis and chlorosis in agroinfiltrated leaves at 7 dpi, which becomes systemic at 10 dpi, severe necrosis/chlorosis develops around 14 dpi and plants die from infection at 18–21 dpi. Whereas infections with CBSV_UCP do not develop symptoms until 10 dpi, when necrosis/chlorosis is observed in agroinfiltrated leaves, which becomes systemic around 14 dpi and more severe at 18–21 dpi but does not result in plant death. Descriptions of severity scores are provided in the “Materials and methods” section. **c** ELISA quantification of CBSV titers during *N. benthamiana* infections with CBSV_Tanza and CBSV_UCP. Viral titers (%) are relative to the positive control sample (100%), supplied with the TAS-ELISA kit (DSMZ). Titers in CBSV_Tanza infections are higher than CBSV_UCP at 7 dpi, peak at 14 dpi and then decrease at 21–28 dpi due to plant death, whereas titers in CBSV_UCP are lower at 7–14 dpi and increase to higher levels later in infection at 21–28 dpi. The cut-off threshold for reliable detection in this assay is 29%

To test whether U/CBSV CP sequences differences are associated with symptom differences, the chimera CBSV_UCP was used to agroinfiltrate 5-week old *N. benthamiana* plants. Infections were performed in three repeat experiments, which produced the following consistent results. *N. benthamiana* agroinfiltrated with CBSV_UCP developed systemic symptoms (Fig. 3). RT-PCR (Fig. S3) and amplicon sequencing (Fig. S11) confirmed the presence of UCBSV Kikombe CP sequence in the upper systemic leaves of CBSV_UCP infections at 10 dpi. This demonstrates that the chimeric virus CBSV_UCP can infect, replicate and move systemically in *N. benthamiana*.

Differences in symptom development were observed during infections with CBSV_UCP compared with unmodified CBSV_Tanza (Fig. 3). At 7 dpi, agroinfiltrated leaves in CBSV_Tanza infections developed necrosis, whereas CBSV_UCP infections developed chlorosis. At 10 dpi, CBSV_Tanza infections developed severe systemic necrosis, whereas no systemic symptoms were present in CBSV_UCP infections. At 14–18 dpi, CBSV_Tanza infections developed severe systemic necrosis that resulted in plant death, whereas CBSV_UCP infections developed mild necrosis in upper systemic leaves, which became more severe at 18–21 dpi but did not result in plant death. Plants were scored for symptom severity throughout infections (Fig. 3). This shows that overall CBSV_UCP infections exhibit both delayed symptom development and a reduction in the severity of necrosis, compared with unmodified CBSV_Tanza infections. Quantification of viral titers during infections revealed that viral titers in unmodified CBSV_Tanza infections are higher than those of CBSV_UCP at 7 dpi, peak at 14 dpi and then decrease at 21–28 dpi due to plant death. Whereas, viral titers in CBSV_UCP infections are lower than CBSV_Tanza at 7–14 dpi and increase to higher levels at 21–28 dpi (Fig. 3). This indicated that CBSV CP is involved with relatively high levels of viral accumulation during early CBSV infection of *N. benthamiana*. To test whether CBSV_UCP is infectious, a mechanical inoculation experiment was performed. *N. benthamiana* plants infected with unmodified CBSV_Tanza or CBSV_UCP were sampled at 14 dpi and material from each was used to mechanically back-inoculate six *N. benthamiana* plants. Symptom development in these mechanical infections was consistent with the first agroinfiltration passage (Fig. S12), demonstrating that differences in symptom development during CBSV_Tanza and CBSV_UCP infections were not dependent on inoculation method.

## Discussion

In this study, GFP was inserted into the CBSV_Tanza IC at two genome positions: between the Ham1—CP (CBSV_GFP1) and between the CP—3′UTR (CBSV_GFP2). This is the first report of a CBSV IC being modified with a marker protein sequence. The localization of CBSV in epidermal and mesophyll cells of *N. benthamiana* is consistent with untagged CBSV localization to epidermal and mesophyll cells and phloem, detected using immune-histochemical staining of CBSD infected cassava [28]. Green fluorescence during CBSV_GFP1/2 infections of *N. benthamiana* appeared to peak at 14 dpi, which may correspond to a peak in CBSV replication, as was identified during untagged CBSV infections (Fig. 3). The detection of green fluorescence in systemic leaf tissues infected with CBSV_GFP1/2 from 7 dpi, is relatively late in infection compared with other viruses; during *N. clevelandii* infections with GFP tagged *Potato virus X* (PVX), green fluorescence was visible in upper systemic leaves at 5 dpi [29] and during *N. tabacum* infections with GUS tagged *Tobacco etch virus* (TEV), GUS was detected in roots at 1 dpi and stems at 2 dpi [30]. The delay in marker gene detection during CBSV_GFP1/2 infections of *N. benthamiana* may be due to inherent differences during CBSV and PVX/TEV infections of *Nicotiana* spp. Alternatively, marker gene insertion may have a greater detrimental effect on CBSV infection mechanisms compared to PVX and TEV. Compared with necrotic CBSV_Tanza infections, CBSV_GFP1/2 infections were asymptomatic, and titers were dramatically lower. A reduction in symptom expression and viral accumulation during infections with tagged viruses has also been reported for *Turnip mosaic virus* (TuMV) [31], PVX [29] and *Lettuce mosaic virus* (LMV) [32]. This is likely to be due to interference of the marker gene with viral infection mechanisms. Selection pressure may then act to delete the marker gene through recombination during viral genome replication. Once a deletion has occurred the wild-type like virus may have a selective advantage over tagged viruses, leading to an increase in the deletants over time [33]. In this study, CBSV genomes containing GFP deletions were detected in 25% of plants infected with CBSV_GFP1/2. Marker gene deletions have also been reported during infections with tagged *Zucchini yellow mosaic virus* (ZYMV) [34], TuMV [31], *Tobacco mosaic virus* (TMV) [35], *Plum pox virus* (PPV) [36] and TEV [37]. There does not appear to be detectable difference in GFP stability during CBSV_GFP1 and CBSV_GFP2 infections, as similar levels of green fluorescence were detected by microscopy and a similar proportion of plants contained deletants. Ideally, marker gene tagged CBSV would be stable through multiple host passages and so optimization may be required. Once these issues have been addressed, tagged U/CBSV ICs will be valuable tools to: characterize viral gene functions, identify whether CBSV and UCBSV co-infect cells during mixed infections and determine vector-binding sites.

In addition to marker gene insertion, the CBSV_Tanza IC was also used to construct a chimera: CBSV_UCP, containing a UCBSV Kikombe CP replacement. The CP region was selected because CBSV and UCBSV CP sequences share low sequence homology at their N’ ends [6], and so it was hypothesized that the CP region may be associated with differences in symptom development during typically necrotic CBSV and mild UCBSV infections of *N. benthamiana*. When used to agroinfiltrate or mechanically inoculate *N. benthamiana*, CBSV_UCP was able to cause systemic infections. As CPs are required for the systemic movement of nearly all plant viruses [20], it seems likely that the UCBSV CP is able to trans-encapsidate the CBSV genome. Trans-encapsidation has been reported during mixed infections with barley yellow dwarf luteoviruses, which results in altered vector specificities [38]. Mixed CBSV and UCBSV cassava infections are relatively common, making up 30–50% of tested infections in Kenya, Tanzania and Uganda [7, 14, 39] and so trans-encapsidation may occur in the field, potentially resulting in altered vector transmission specificity and or/efficiency. The presence of the DAG motif in CBSV CPs has led to speculation that aphids in addition to whitefly may transmit CBSVs [17]. The CBSV_UCP could, therefore, be used to further investigate U/CBSV vector transmission specificity and efficiency, which are currently poorly understood.

In terms of symptom development, systemic necrosis developed during *N. benthamiana* infections with CBSV_UCP, indicating that additional genome regions other than CP may be associated with necrosis development during CBSV infections. However there were distinct changes in the severity and timing of symptom development. Whereas *N. benthamiana* infected with CBSV_Tanza develop severe systemic necrosis by 14 dpi, CBSV_UCP infections only develop mild necrosis by 18 dpi. Therefore, the CBSV CP may be associated with high levels of necrosis during early infection.

Viral titers are also lower during early CBSV_UCP infections, compared with unmodified CBSV_Tanza. This indicates that, compared with the UCBSV CP, the CBSV CP may enable higher viral accumulation during early infection. Alternatively, lower CBSV_UCP titers during early infection may be due to: (1) a reduced efficiency of proteolytic cleavage of the UCBSV CP from the CBSV polyprotein, (2) a reduced ability for the UCBSV CP to interact with CBSV_Tanza proteins and/or (3) a reduced ability for the UCBSV CP to trans-encapsidate the CBSV genome for movement. It is also possible that any modifications to the CBSV genome results in reduced viral accumulation and so alteres symptom expression.

We performed phylogenetic analysis of U/CBSV CP sequences, which indicated that the CBSV_Tanza and UCBSV Kikombe CP sequences cluster within their separate species clades, and so should be relatively representative of CBSV and UCBSV. However, U/CBSVs are highly diverse [40] and different CBSV and UCBSV CPs are likely to function differently and have different symptom associations. Therefore, future work should involve a range of chimeras for a variety of CBSV and UCBSV isolates, which would further validate characterization of symptom determinants. It is also important to note that there may be different U/CBSV sequences associated with symptom development during infections of *N. benthamiana* and cassava and so U/CBSV ICs should ideally be tested in cassava.

In conclusion, the CBSV_Tanza IC manipulations reported in this study have enabled visualization of CBSV replication in planta and provided initial insights into the viral sequences associated with symptom development and accumulation. These manipulations represent important progress in understanding the fundamental biology of U/CBSVs and how they cause devastating food insecurity. Ultimately, this understanding should inform vital CBSD control strategies, which are urgently needed.

## Electronic supplementary material

Below is the link to the electronic supplementary material.

Supplementary material 1 (DOCX 3208 kb)
