# Supplementary material for: Utilization of infectious clones to visualize Cassava brown streak virus replication in planta and gain insights into symptom development
Source: Virus Genes. 2019 Aug 6;55(6):825–33. doi: 10.1007/s11262-019-01697-5 (PMC6831539; doi:10.1007/s11262-019-01697-5)
Supplement: Supplementary file 1 — Supplementary material 1 (DOCX 3208 kb) [file 11262_2019_1697_MOESM1_ESM.docx]

Supplementary material: Utilization of infectious clones to visualize *Cassava brown streak virus* replication in planta and gain insights into symptom development

Authors: Katie R. Tomlinson^1^, Susan E. Seal^2^, Andy M. Bailey^1^ and Gary D. Foster^1^

1. School of Biological Sciences, University of Bristol, 24 Tyndall Ave, Bristol BS8 1TQ, UK.

2. Natural Resources Institute, University of Greenwich, Central Avenue, Chatham Maritime, UK.

Corresponding author: Prof. Gary D. Foster ([gary.foster@bristol.ac.uk](mailto:gary.foster@bristol.ac.uk); +44 (0) 117 39 41178; ORCiD: 0000-0002-1979-1397).

**A)**


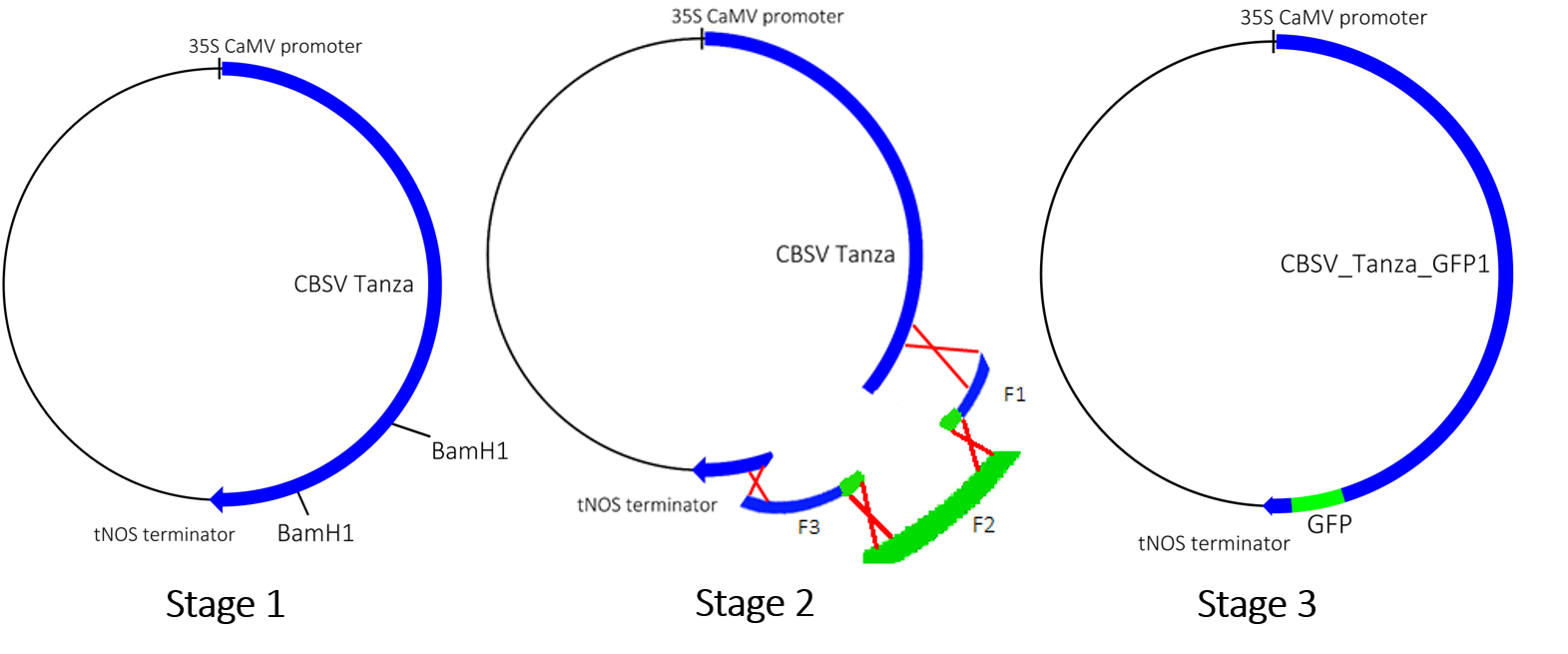


**B)**


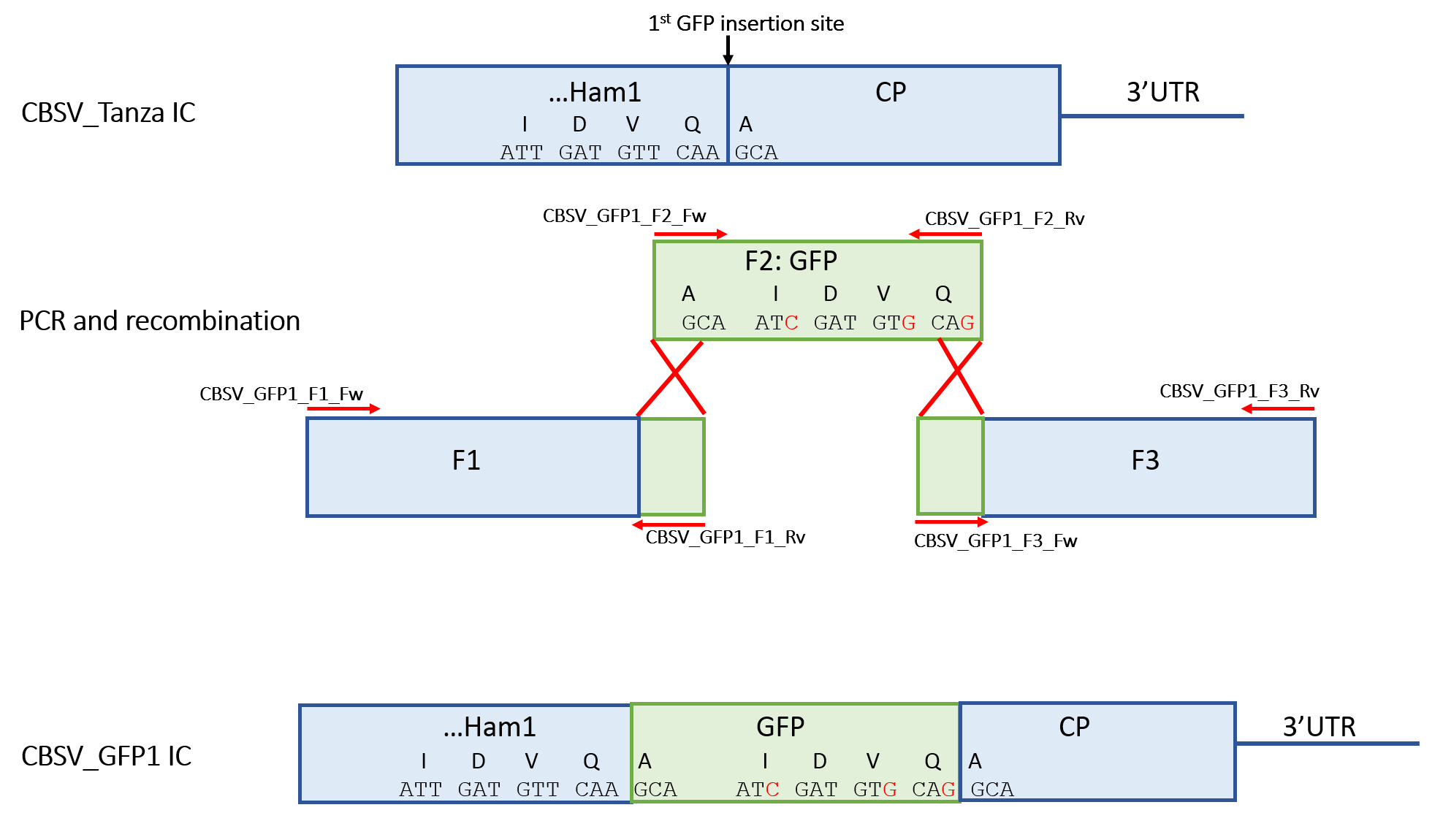


**C)**


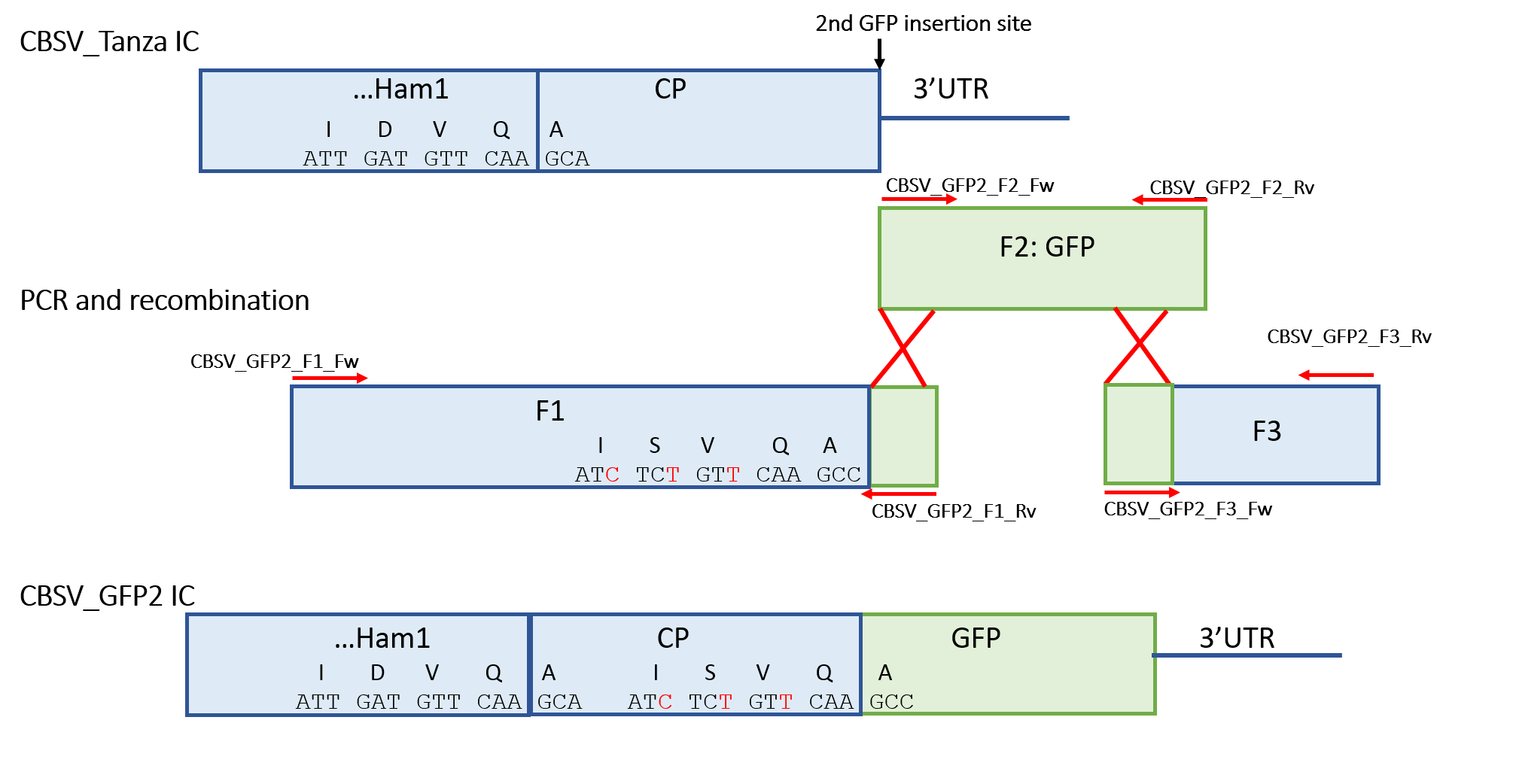


Figure S1: Schematics for the construction the CBSV_GFP1/2 infectious clones (IC) using homologous yeast recombination. **a** To construct CBSV_GFP1/2, the CBSV_Tanza IC was digested with *Bam*H1, which generates fragments of 18.6 Kb and 1.7 Kb (stage 1). The 18.6 Kb fragment and three overlapping PCR fragments: F1 – 3, were then transformed a recombined in yeast (stage 2) and yeast plasmids were rescued into *E. coli* to form CBSV_GFP1/2 (stage 3). Schematics for the design of PCR fragments are shown for the construction of CBSV_GFP1 (**b**) and CBSV_GFP2 (**c**). PCR products were designed so that fragment 1 (F1): at the 5’ end contains homologous sequence with the CBSV_Tanza IC from before the first *Bam*H1 restriction site and at the 3’ end contains homologous sequence with fragment 2 (F2); F2: encodes the GFP sequence; Fragment 3 (F3): at the 5’ end contains homologous sequence with GFP, it then encodes remaining CBSV sequence and at the 3’ end it contains homologous sequence with CBSV_Tanza IC from after the second *Bam*H1 restriction site. To enable release of the GFP peptide from the CBSV polyprotein, sequence encoding the Ham1 – CP proteolytic cleavage site: I-D-V-Q-/-A was also cloned onto the 3’ end of the GFP sequence in the CBSV_GFP1 IC. Whereas in CBSV_GFP2, the NIa - NIb cleavage site: I-S-V-Q-/-A was added to the 5’ of the GFP sequence and no cleavage site was necessary at the 3’ end as GFP is the last peptide in the polyprotein. To reduce nucleotide similarity between cleavage site sequences, the third base in the isoleucine, valine and glutamine codons was changed to an alternative nucleotide (red). Primers used in the amplification of PCR products are provided in Table S1.

**A)**


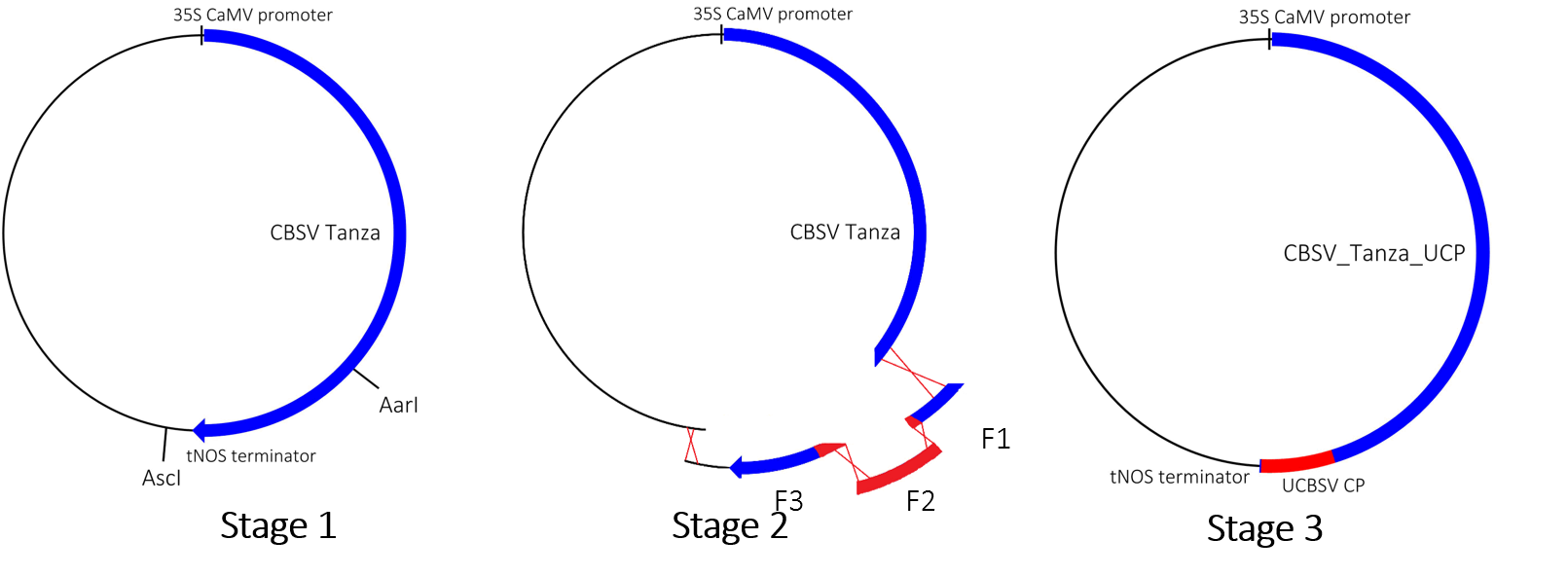


**B)**


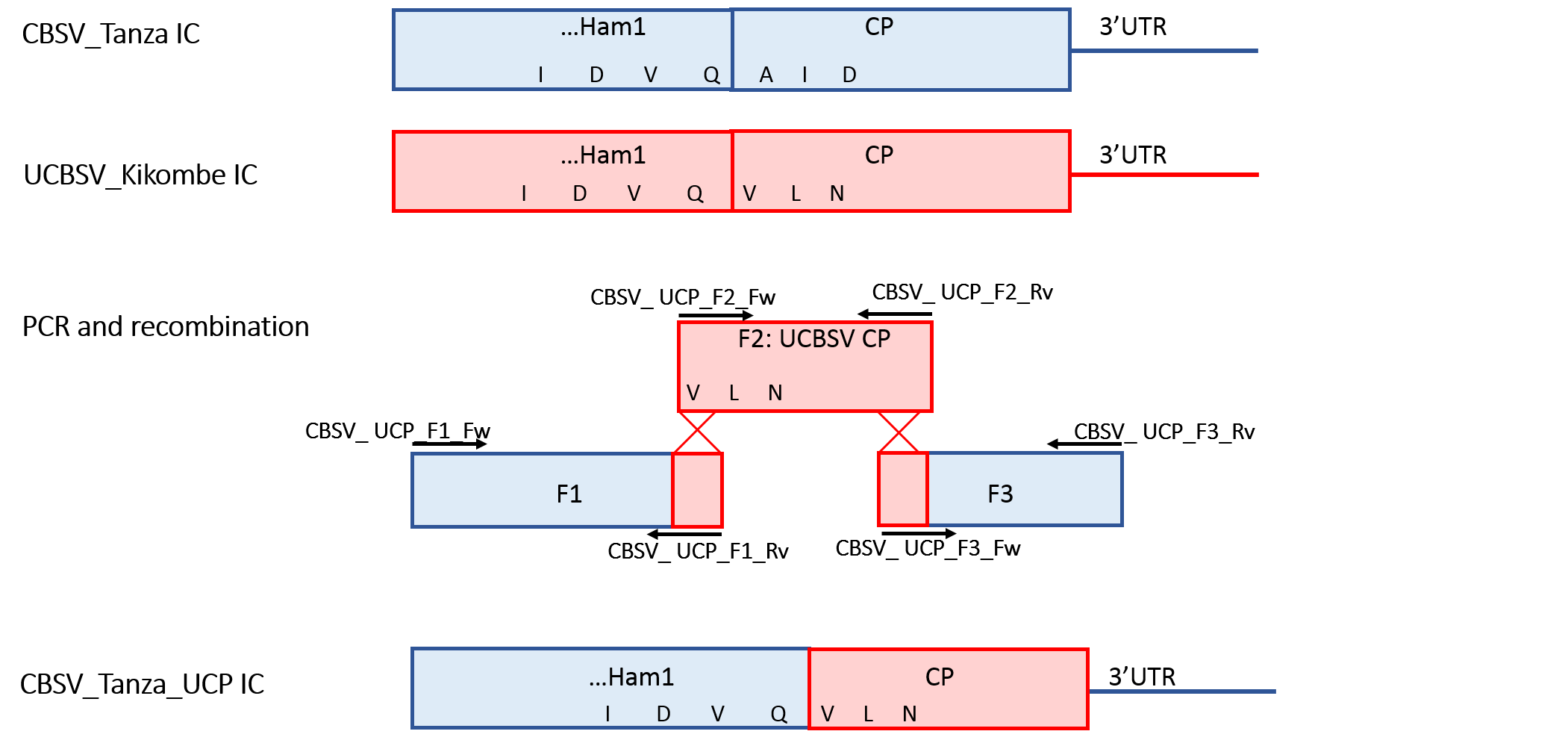


Figure S2: Schematics for the construction the CBSV_UCP infectious clone (IC) using homologous yeast recombination. **a** To construct CBSV_UCP, the CBSV_Tanza IC was digested with *Aar*I and *Asc*I, which generates fragments of 16.9 Kb and 3.2 Kb (stage 1). The 16.9 Kb fragment and PCR fragments: F1 - F3 were then recombined in yeast (stage 2) and yeast plasmids were rescued into *E. coli* to form the CBSV_Tanza_UCP IC. **b** Schematic for the design of PCR fragments are shown for the construction of CBSV_UCP. PCR fragment 1 (F1): at the 5’ end contains homologous sequence from before the *Aar*I restriction site in the CBSV_Tanza IC plasmid, it then encodes CBSV_Tanza sequence from NIb to the end of Ham1 and at the 3’ end it contains 30 bp of homologous sequence with fragment 2 (F2); F2: encodes the UCBSV Kikombe CP sequence and Fragment 3 (F3): at the 3’ end contains homologous sequence with F2, it then encodes CBSV 3’UTR sequence and at the 3’ end it contains homologous sequence with CBSV_Tanza IC from after the *Asc*I restriction site. The proteolytic cleavage sequence: I-D-V-Q-/-V was inserted between the CBSV Ham1 and UCBSV CP peptides, which should enable release of the UCBSV CP from the CBSV polyprotein. Primers used in the amplification of PCR products are provided in Table S1.

**A) B)**

**
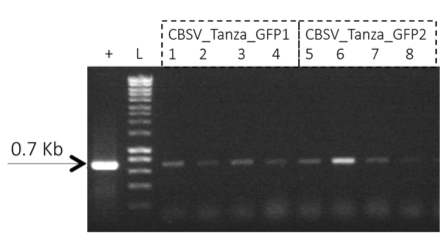
**
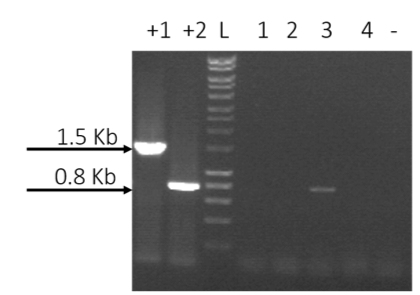


**C) D)**


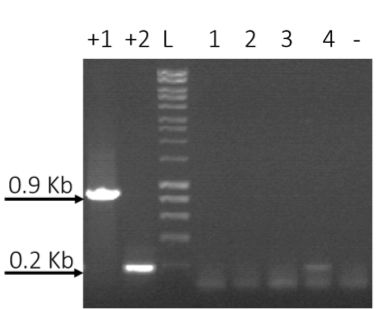

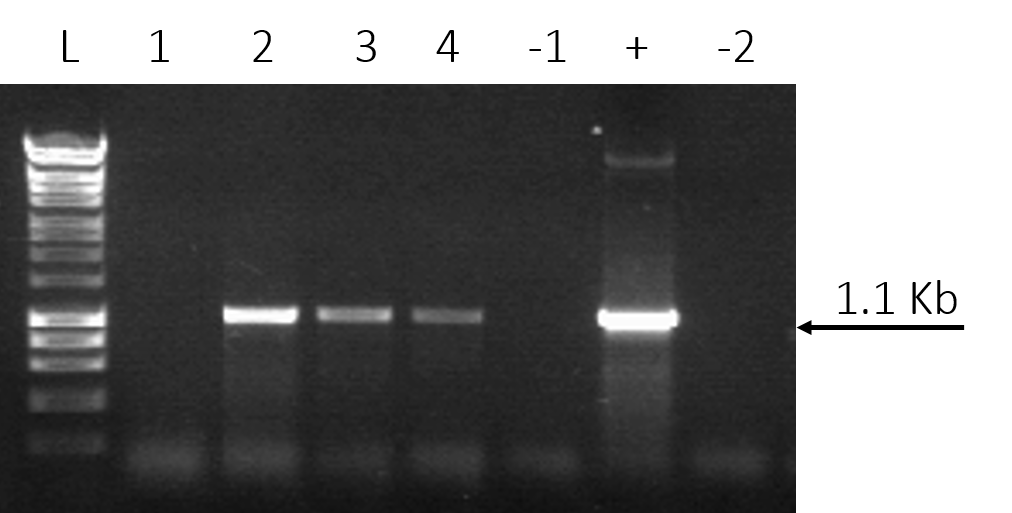


Figure S3: RT-PCR detection of CBSV_GFP1/2 infections of *N. benthamiana*. **a** Detection of the GFP fragment (0.7 Kb) in upper systemic leaves of *N. benthamiana plants* agroinfiltrated with CBSV_GFP1 (lanes 1 – 4) or CBSV_ GFP2 (lanes 5 – 8) at 10 dpi. Amplification of the GFP fragment occurred in the positive control PCR when the CBSV_GFP1 IC was used as template (+). PCR was performed with the CBSV_GFP1_F2_Fw/Rv (Table S1), which target the GFP fragment. **b** Detection of viral transcripts containing a GFP deletion in one (lane 3) of the four *N. benthamiana* plants (lanes 1 – 4) agroinfiltrated with CBSV_GFP1 at 15 dpi. PCR was performed with CBSV_GFP1_del1_Fw/Rv primers (Table S3), which target the GFP insertion site between the Ham1 and CP. Amplification products of 1.5 Kb occurred in the positive control PCRs: when the CBSV_GFP1 IC plasmid was used as template (+1) and amplification of 0.8 Kb occurred when the CBSV_Tanza IC plasmid was used as template (+2). No amplification occurred with cDNA from a non-inoculated plant (-). **c** Detection of viral transcripts containing GFP deletions in one (lane 4) of four *N. benthamiana* plants agroinfiltrated with CBSV_GFP2 at 15 dpi. PCR was performed with CBSV_GFP2_del2_Fw/Rv primers (Table S3), which target the GFP insertion site between the CP and 3’UTR. Amplification of 0.9 Kb occurred in the positive control PCRs: when CBSV_GFP2 IC plasmid was used as template (+1) and amplification of 0.2 Kb occurred when the CBSV_Tanza IC plasmid was used as template (+2). No amplification occurred with cDNA from a non-inoculated plant (-). **d** Detection of UCBSV Kikombe CP in systemic leaf material sampled from three *N. benthamiana* plants (lanes 2 – 4) infected with the CBSV_UCP IC, at 14 dpi. The CBSV_ UCP_F2_Fw/Rv primers (Table S1) were used, which specifically amplify the UCBSV Kikombe CP fragment = 1.1 Kb. Amplification of 1.1 Kb occurred in the positive control PCR when CBSV_Tanza_UCP IC plasmid was used as template (+). No amplification occurred from a non-inoculated plant (-1) or when no template was added to the PCR (-2).

Figure S4: Sanger sequence read generated from a reaction with the pJET2.1 Fwd primer and a pJET2.1 plasmid (Thermo Fisher Scientific) containing a cloned GFP RT-PCR amplicon, amplified from the upper systemic leaf material from an *N. benthamiana* plant infected with CBSV_Tanza_GFP1 at 10 dpi. The sequence aligns to the CBSV_Tanza_GFP1 map at positions 9139 – 9858 bp, which corresponds to the GFP insertion at positions 9139 – 9867 bp.

Figure S5: Sanger sequence read generated from a reaction with the pJET2.1 Fwd primer and a pJET2.1 plasmid (Thermo Fisher Scientific) containing a cloned GFP RT-PCR amplicon, amplified from the upper systemic leaf material from an *N. benthamiana* plant infected with CBSV_Tanza_GFP2 at 10 dpi. The sequence aligns to the CBSV_Tanza_GFP2 map at positions 10288 – 10958 bp, which corresponds to the GFP insertion at positions 10285 – 10999 bp.


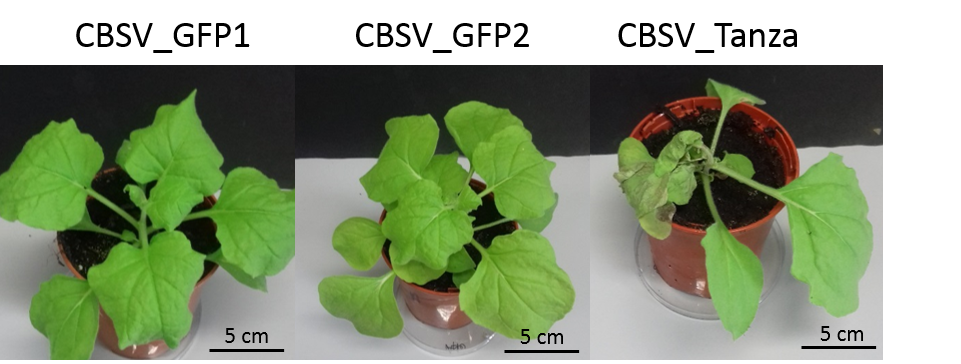


Figure S6: *Nicotiana benthamiana* infected with CBSV_Tanza_GFP1 and CBSV_GFP2 were symptomless, compared with highly necrotic infections with the unmodified CBSV_Tanza IC, at 14 dpi.

Figure S7: QPCR quantification of relative viral transcript abundance in systemic leaves of *N. benthamiana* plants agroinfiltrated with CBSV_Tanza, compared with CBSV_Tanza_GFP1 and CBSV_Tanza_GFP2 at 15 dpi. Transcript abundance of CBSV_GFP1 and CBSV_Tanza_GFP2 is dramatically reduced compared with unmodified CBSV_Tanza.

Figure S8: Sanger sequence read generated from a reaction using the pJET2.1 Fwd primer and a pJET2.1 plasmid (Thermo Fisher Scientific) containing a cloned RT-PCR amplicon, amplified from the upper systemic leaf material from an *N. benthamiana* plant infected with CBSV_Tanza_GFP1 at 15 dpi. The sequence aligns to the CBSV_Tanza_GFP1 map at positions 8920 – 9138 bp, which corresponds to the end of the CBSV Ham1 sequence, the RT-PCR fragment is then missing the GFP sequence between positions 9139 – 9867 bp and realigns at positions 9868 – 10149 bp corresponding to the start of the CBSV CP sequence. This demonstrates detection complete GFP deletion.

Figure S9: Sanger sequence read generated from a reaction using the pJET2.1 Fwd primer and a pJET2.1 plasmid (Thermo Fisher Scientific) containing a cloned RT-PCR amplicon, amplified from the upper systemic leaf material from an *N. benthamiana* plant infected with CBSV_Tanza_GFP2 at 15 dpi. The sequence aligns to the CBSV_Tanza_GFP2 map at positions 10080 – 10284 bp, which corresponds to the end of the CBSV CP sequence, the RT-PCR fragment is then missing the GFP sequence between positions 10285 – 10999 bp and realigns at positions 11000 – 11057 bp corresponding to the CBSV 3’UTR sequence. This demonstrates detection complete GFP deletion.

Figure S10: Phylogenetic tree built using 19 CBSV and 23 UCBSV CP amino acids sequences. The CBSV Tanza (grey circle) and UCBSV Kikombe CP (black circle) cluster within their respective CBSV and UCBSV species clades and so should be relatively representative of CBSV and UCBSV. The tree was inferred using the Maximum Likelihood method based on the Whelan and Goldman model, with 250 bootstrap replicates. The tree with the highest log likelihood (-3041.4173) is shown. Bootstrap values are provided for each branch. The tree is rooted using the out-group CP sequence from a CBSV isolate (NCBI:AY007597). Sequences were obtained from NCBI databases; accession numbers for each sequence are provided in the tree. The tree is drawn to scale where 0.05 = unit of evolutionary distance in terms of number of amino acids substitutions per site. Analyses were conducted in MEGA6.

Figure S11: Sanger sequence read generated from a reaction using the pJET2.1 Fwd primer and a pJET2.1 plasmid (Thermo Fisher Scientific) containing a cloned RT-PCR amplicon, amplified from the upper systemic leaf material from an *N. benthamiana* plant infected with CBSV_UCP at 14 dpi. The sequence aligns to the CBSV_UCP map at positions 10091 – 10231 bp, which corresponds to UCBSV Kikombe CP sequence at positions 9139 – 10242 bp. This confirms that CBSV_Tanza_UCP can systemically infect *N. benthamiana.*


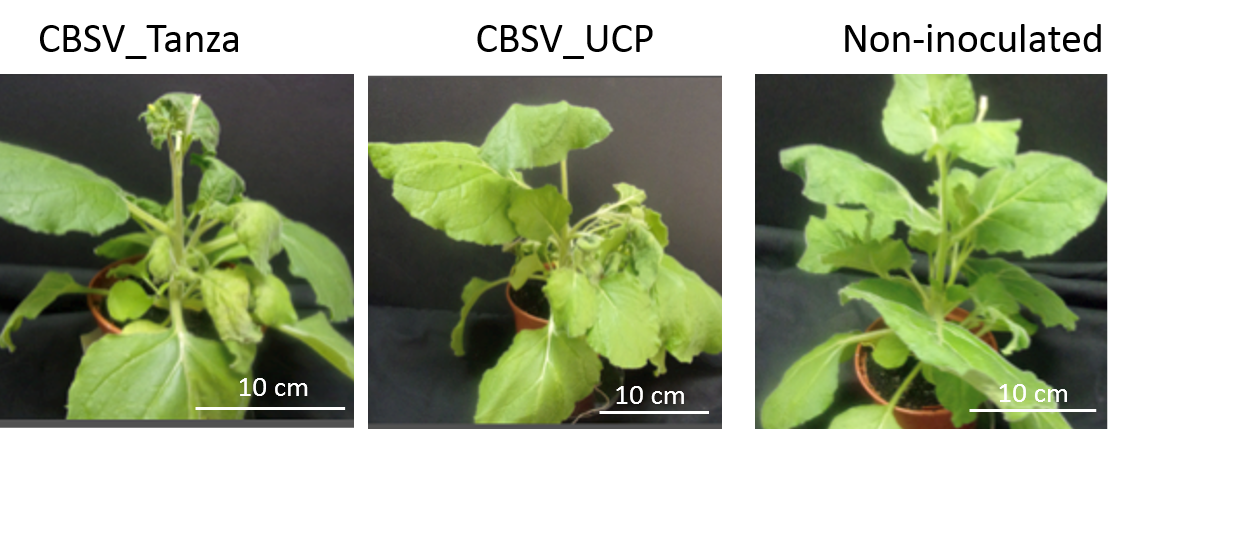


Figure S12: Symptom development during infections of *N. benthamiana* mechanically back inoculated with CBSV_Tanza or CBSV_UCP infected material, compared to symptomless non-inoculated plant, at 18 dpi. Plants infected with CBSV_Tanza developed severe systemic chlorosis, necrosis and wilting, whereas plants infected with CBSV_UCP developed milder systemic necrosis and wilting. Symptom development in this second passage was consistent with the first passage, where plants were agroinfiltrated

Table S1: Primers used to amplify PCR fragments used in the construction of the CBSV_GFP1, CBSV_GFP2 and CBSV_UCP infectious clones. Insert sequences (GFP/UCBSV CP) are shown in *itallics* and sequence encoding proteolytic cleavage sites are shown in bold.

| **Infectious clone** | **Primer** | **5’ – 3’ sequence** | **Size (bp)** | **Target** |
| --- | --- | --- | --- | --- |
| CBSV_GFP1 | CBSV_GFP1_F1_Fw | GGAAGGGTTTGCTGAAATATAATGAACCTG | 2021 | CBSV Ham1 – GFP |
|  | CBSV_GFP1_F1_Rv | *CTCCAGTGAAAAGTTCTTCTCCTTTACTCAT***TGCTTGAACATCAAT**AAAGAAATCACGATCA |  |  |
|  | CBSV_GFP1_F2_Fw | **GCA***ATGAGTAAAGGAGAAGAACTTTTCACTGGAGTT* | 723 | GFP |
|  | CBSV_GFP1_F2_Rv | **CTGCACATCGAT***TTTGTATAGTTCATCCATGCCATG* |  |  |
|  | CBSV_GFP1_F3_Fw | *GATGAACTATACAAA***ATCGATGTGCAGGCA**  ATTGACAAGGATGAGATTGAAGCTGAAA | 1711 | GFP - CBSV CP |
|  | CBSV_GFP1_F3_Rv | GGCTGGCTGGTGGCAGGATATATTGTGGTGTAAA |  |  |
| CBSV_GFP2 | CBSV_GFP2_F1_Fw | GGAAGGGTTTGCTGAAATATAATGAACCTG | 3154 | CBSV CP – GFP |
|  | CBSV_GFP2_F1_Rv | *GTGAAAAGTTCTTCTCCTTT***ACTGGCTTGAACAGAGAT**TTCAATAGCAGCACCAGC |  |  |
|  | CBSV_GFP2_F2_Fw | **GCC***AGTAAAGGAGAAGAACTTTTCACTGGAGTTGTC* | 723 | GFP |
|  | CBSV_GFP2_F2_Rv | *CAAATTAATTTTTGTATAGTTCATCCATGCCATGTGTAATCC* |  |  |
|  | CBSV_GFP2_F3_Fw | *GATTACACATGGCATGGATGAACTATACAAAA*ATTAATTTGAAAGTCAAGTTTGGTGGAG | 587 | GFP – CBSV 3’UTR |
|  | CBSV_GFP2_F3_Rv | GGAAGGGTTTGCTGAAATATAATGAACCTG |  |  |
| CBSV_UCP | CBSV_UCP_F1_Fw | AGAGGAATTCATTGTGTCAGTGCGTGAAAGT | 1790 | CBSV NIb |
|  | CBSV_ UCP_F1_Rv | *GTCTCTGCTTCTATCTCCTCCTGATTTAAG***ACTTGAACATCAAT**AAAGAAATCA |  |  |
|  | CBSV_ UCP_F2_Fw | **GTC***TTAAATCAGGAGGAGATAGAAGCA* | 1103 | UCBSV CP |
|  | CBSV_UCP_F2_Rv | *TTATTCAATTGCGGCACCACTGTAACTGTGTCTGTTTGCACTAAC* |  |  |
|  | CBSV_UCP_F3_Fw | *TTACAGTGGTGCCGCAATTGAATAA*ATTAATTTGAAAGT | 572 | CBSV 3’UTR |
|  | CBSV_UCP_F3_Rv | GGTGGCAGGATATATTGT |  |  |

Table S2: Primer sequences used in qPCR.

| **Primer** | **Sequence 5’ – 3’** | **Amplicon size (bp)** | **Target** |
| --- | --- | --- | --- |
| qPCR_CBSV_CP_Fw | ACTTCCTAGCCGAAGCACAA | 163 | CBSV coat protein |
| qPCR_CBSV_CP_Rv | GCACTAACATCCCGCGTAGT |  |  |
| qPCR_Fbox_Fw | GGCACTCACAAACGTCTATTTC | 127 | *N. benthamiana* F-BOX (TAIR: At5g15710) |
| qPCR_Fbox_Rv | ACCTGGGAGGCATCCTGCTTAT |  |  |

Table S3: Primer sequences used in RT-PCR to detect the presence or absence of GFP in *N. benthamiana* infections with the CBSV_Tanza and CBSV_Tanza_GFP1/2 ICs.

| **Primer name** | **Primer sequence** | **Amplicon size (bp)** |
| --- | --- | --- |
| CBSV_GFP1_del1_Fw | GTGAGATTGTGATGCCACGCGGACCAAATT | CBSV_Tanza = 804  CBSV_Tanza_GFP1 = 1536 |
| CBSV_GFP1_del_Rv | CAACTTCTCCTTCATCAGGTTCTCCACATGGCCTCTTAC |  |
| CBSV_GFP2_del2_Fw | GCTTTAGCTGCTCCTGGTGATGATAATAAT | CBSV_Tanza = 914  CBSV_Tanza_GFP2 = 190 |
| CBSV_GFP2_de2l_Rv | GAAAGGGTTCCTGAATATATCTTGGCTTCA |  |
